# Supplementary material for: Increase in the Bioactive Potential of Olive Pomace Oil after Ultrasound-Assisted Maceration
Source: Foods. 2023 May 26;12(11):2157. doi: 10.3390/foods12112157 (PMC10252974; doi:10.3390/foods12112157)
Supplement: Supplementary file 1 [file foods-12-02157-s001.zip › foods-2401032-supplementary.pdf]

Table S1

Quality parameters of each experiment of the central composite design for the ultrasound-assisted maceration (UAM) of rosemary in olive pomace oil.

Experiments	FFA[a] (% oleic acid)	PV[a] ($meqO_2.kg^{-1}$)	K_{232}[a]	K_{270}[a]	ΔK[a]
**1**	0.115	2.976	4.033	1.378	0.129
**2**	0.103	3.498	4.220	1.414	0.127
**3**	0.102	2.987	4.154	1.387	0.128
**4**	0.089	3.496	4.287	1.450	0.129
**5**	0.115	4.995	4.669	1.415	0.125
**6**	0.103	4.417	4.614	1.456	0.126
**7**	0.116	4.990	3.851	1.402	0.121
**8**	0.103	5.982	3.502	1.317	0.118
**9**	0.089	3.961	3.212	1.261	0.118
**10**	0.103	6.974	3.801	1.452	0.125
**11**	0.102	4.943	3.961	1.350	0.121
**12**	0.102	4.460	2.834	1.463	0.115
**13**	0.075	2.966	3.404	1.327	0.118
**14**	0.076	3.474	3.790	1.354	0.122
**15**	0.103	4.466	2.973	1.220	0.114
**16**	0.090	3.419	3.440	1.284	0.118
**17**	0.076	3.485	3.898	1.324	0.119
**18**	0.115	3.476	3.510	1.380	0.121

[a] FFA = free fatty acids; PV = peroxide value; K_{232}, K_{270} and ΔK = specific extinction coefficients

Table S2

Total phenolic content, antioxidant capacity (Oxygen Radical Absorbance Capacity, ORAC), polar compounds, and Rancimat of each central composite design experiment for rosemary's ultrasound-assisted maceration (UAM) in olive pomace oil.

Experiments	Total phenolic content (mg GAE.kg^{-1}) [a]	Antioxidant capacity (ORAC, µmol TE.g^{-1}) [a]	Polar compounds (%TPM)	Rancimat	
				Induction time (h)	Stability time (h)
**1**	13.418	1.30	8.00	1.42	1.82
**2**	14.944	2.87	6.50	1.68	1.96
**3**	15.305	3.53	7.00	1.66	2.02
**4**	10.566	1.11	7.50	1.52	1.87
**5**	17.233	1.57	7.50	1.37	1.79
**6**	13.337	1.44	8.00	1.45	1.79
**7**	14.863	2.34	8.00	1.52	1.85
**8**	14.863	2.46	7.00	1.40	1.72
**9**	12.695	2.31	8.00	1.48	1.76
**10**	12.775	1.20	8.00	1.31	1.67
**11**	11.129	1.27	7.50	1.39	1.81
**12**	15.386	1.92	7.50	1.50	1.81
**13**	13.016	1.22	8.00	1.44	1.73
**14**	13.458	0.76	6.50	1.38	1.72
**15**	13.779	0.27	7.50	1.28	1.61
**16**	13.137	0.28	8.00	1.35	1.62
**17**	13.659	1.29	8.50	1.44	1.74
**18**	10.647	0.76	8.50	1.49	1.76

[a] GAE = gallic acid equivalents; TE = Trolox equivalents.

Table S3

Volatile compounds of each experiment of the central composite design for the ultrasound-assisted maceration (UAM) of rosemary in olive pomace oil.

Volatile compounds (area x 10^4)	Experiments																	
	**1**	**2**	**3**	**4**	**5**	**6**	**7**	**8**	**9**	**10**	**11**	**12**	**13**	**14**	**15**	**16**	**17**	**18**
**α-pinene**	42.53	46.61	96.83	81.18	46.28	55.67	74.09	78.13	64.68	83.37	44.04	66.86	70.80	70.80	83.99	88.50	77.19	58.08
**camphene**	0.42	nd [a]	nd	3.91	1.24	0.86	0.89	nd	0.81	nd	nd	nd	nd	nd	nd	nd	0.38	0.77
**camphor**	0.51	nd	0.34	4.27	nd	nd	nd	nd	nd	nd	nd	0.30	nd	nd	nd	nd	nd	nd
**δ-terpineol**	nd	nd	nd	nd	6.48	5.03	2.27	0.64	1.08	2.29	0.78	2.90	2.70	2.70	0.77	0.94	0.68	0.53
**hexanal**	0.25	0.18	0.36	5.56	2.49	1.64	1.91	nd	1.58	0.40	0.30	0.34	nd	nd	nd	nd	0.35	0.62
**α-terpinolene**	0.38	nd	0.47	2.29	1.44	0.82	0.99	nd	0.88	0.31	nd	0.23	0.24	0.24	0.27	0.19	0.34	0.57
**octanal**	1.10	0.24	0.82	8.47	2.91	1.44	1.64	0.28	1.58	0.45	nd	0.39	0.24	0.24	0.27	0.30	0.56	1.42
**thuja-2,4(10)-diene**	2.86	8.56	13.43	7.16	7.72	5.98	10.95	4.89	7.68	7.54	3.93	7.44	5.14	5.14	8.25	5.63	7.04	6.65
**L-limonene**	nd	nd	0.33	2.84	0.45	0.35	0.44	nd	0.38	0.18	nd	0.22	nd	nd	0.19	nd	0.19	nd
**eucalyptol**	5.58	4.59	9.73	10.47	5.64	8.30	9.82	5.45	7.89	9.42	5.47	7.41	7.84	7.84	10.47	10.33	8.17	7.35
***p*-cymene**	0.72	0.35	0.79	9.20	5.50	3.11	3.63	0.49	3.31	0.70	0.55	0.59	0.53	0.53	0.62	0.63	0.84	1.19
**4-methyl-3-penten-2-one**	10.48	7.54	20.52	21.52	11.57	14.57	19.99	13.53	14.72	19.96	nd	12.94	14.99	14.99	21.67	20.76	16.35	13.32
**6-methyl-5-hepten-2-one**	nd	nd	nd	1.98	0.16	nd	nd	nd	nd	nd	nd	nd	nd	nd	nd	nd	nd	nd
**hexanol**	0.77	0.17	0.72	2.97	0.82	0.58	0.34	0.78	0.36	nd	nd	0.62	0.19	0.19	0.15	0.34	nd	0.21
***(Z)*-3-hexen-1-ol**	nd	0.81	nd	1.58	nd	nd	nd	nd	0.39	nd	nd	nd	nd	nd	nd	nd	nd	nd
**acetic acid**	0.77	nd	0.64	8.21	0.70	0.62	0.33	nd	0.72	nd	0.20	0.51	nd	nd	nd	nd	nd	nd
**estragole**	3.14	nd	2.35	52.66	nd	4.38	3.38	1.73	2.40	3.06	nd	3.13	2.07	nd	1.77	3.86	1.67	1.19

[a] nd: not detected (zero was used as the area for the non-detected compounds during the model's calculation).

Table S4

Quality parameters of each central composite design experiment for basil's ultrasound-assisted maceration (UAM) in olive pomace oil.

Experiments	FFA[a] (% oleic acid)	PV[a] ($meqO_2.kg^{-1}$)	K_{232}[a]	K_{270}[a]	ΔK[a]
**1**	0.103	3.451	3.451	3.486	0.125
**2**	0.063	2.987	3.520	1.477	0.459
**3**	0.103	3.463	3.757	1.404	0.125
**4**	0.089	2.982	4.089	1.388	0.134
**5**	0.076	3.494	4.088	1.464	0.126
**6**	0.076	3.967	4.609	1.625	0.123
**7**	0.089	3.457	4.950	1.544	0.126
**8**	0.076	3.988	3.592	1.384	0.120
**9**	0.076	5.487	3.548	1.404	0.131
**10**	0.089	3.982	2.832	1.305	0.115
**11**	0.075	3.994	3.538	1.342	0.115
**12**	0.076	3.954	4.075	1.400	0.126
**13**	0.075	3.964	3.815	1.275	0.122
**14**	0.103	3.978	4.303	1.389	0.119
**15**	0.089	3.498	3.767	1.313	0.121
**16**	0.076	3.459	3.951	1.415	0.121
**17**	0.116	4.438	3.330	1.481	0.121
**18**	0.102	2.979	3.236	1.517	0.112

[a] FFA = free fatty acids; PV = peroxide value; K_{232}, K_{270} and ΔK = specific extinction coefficients

Table S5

Total phenolic content, antioxidant capacity (Oxygen Radical Absorbance Capacity, ORAC), polar compounds, and Rancimat of each central composite design experiment for basil's ultrasound-assisted maceration (UAM) in olive pomace oil.

Experiments	Total phenolic content (mg GAE.kg^{-1}) [a]	Antioxidant capacity (ORAC, µmol TE.g^{-1}) [a]	Polar compounds (%TPM) [a]	Rancimat	
				Induction time (h)	Stability time (h)
**1**	10.647	0.43	6.50	1.18	1.32
**2**	9.602	0.43	7.50	0.04	0.88
**3**	11.289	0.80	7.00	0.86	1.21
**4**	9.281	0.01	5.50	1.07	1.39
**5**	10.406	0.09	7.50	0.79	1.10
**6**	10.687	0.53	7.50	1.11	1.55
**7**	12.614	1.19	8.00	1.01	1.24
**8**	9.080	0.41	9.50	0.04	1.34
**9**	11.851	1.01	10.00	1.10	1.45
**10**	12.213	0.98	6.50	1.10	1.39
**11**	9.683	0.41	6.00	1.09	1.35
**12**	9.482	0.50	6.50	1.10	1.36
**13**	10.687	0.31	6.00	0.96	1.36
**14**	11.048	0.19	9.00	1.13	1.42
**15**	9.120	0.44	7.00	1.11	1.37
**16**	10.446	0.46	6.50	1.13	1.51
**17**	11.048	0.51	7.00	1.10	1.35
**18**	10.205	0.31	8.50	1.00	1.31

[a] GAE = gallic acid equivalents; TE = Trolox equivalents, TPM = total polar materials.

Tabela S6

Volatile compounds of each experiment of the central composite design for the ultrasound-assisted maceration (UAM) of basil in olive pomace oil.

Volatile compounds (area × 10^4)	Experiments																	
	1	2	3	4	5	6	7	8	9	10	11	12	13	14	15	16	17	18
**L-limonene**	0.70	1.04	1.23	0.83	1.27	1.08	2.60	1.56	1.17	1.38	0.86	0.98	0.82	1.70	1.36	0.94	0.99	1.61
**D-limonene**	nd [a]	nd	nd	nd	nd	nd	0.52	0.31	0.22	0.21	nd	nd	nd	0.28	0.21	0.21	nd	0.30
**sabinene**	0.23	0.33	0.10	0.23	0.10	0.12	nd	0.03	0.38	0.07	0.11	0.48	0.25	0.16	0.38	0.05	nd	0.06
**L-linalool**	nd	nd	nd	nd	nd	0.22	nd	nd	nd	nd	nd	nd	nd	nd	nd	nd	0.46	nd
**β-phellandrene**	nd	0.14	0.21	nd	0.29	0.23	0.52	0.36	0.12	0.29	0.21	nd	0.20	0.28	0.28	0.20	0.19	0.32
**hexanal**	0.66	0.68	0.79	0.49	0.77	0.85	nd	nd	0.63	0.24	0.42	0.87	0.56	0.41	0.62	0.34	0.20	0.33
**4-pentenal**	nd	nd	0.91	0.41	0.59	0.62	0.42	0.44	0.50	nd	0.41	0.62	0.48	0.34	0.34	0.67	nd	0.44
**1-decene**	0.54	0.62	1.07	0.57	0.39	0.99	1.10	0.52	0.48	0.69	0.81	0.91	0.80	0.70	0.99	0.58	0.41	0.69
***p*-menth-1-ene**	0.30	0.34	0.67	0.18	0.58	0.72	0.16	0.23	0.49	nd	0.21	0.81	0.35	0.18	0.33	nd	nd	nd
**methyl isobutyl ketone**	17.71	28.91	31.98	26.91	30.73	28.43	53.62	39.67	35.43	35.24	26.16	32.39	24.31	51.56	36.04	28.79	28.68	42.14
**6-methyl-5-hepten-2-one**	0.32	0.29	1.41	0.25	1.04	1.89	0.81	0.48	0.25	0.28	0.20	0.56	0.27	0.24	0.31	0.51	0.18	0.35
**1-pentanol**	nd	0.29	0.30	0.27	nd	0.37	0.82	0.40	0.29	0.35	0.27	0.01	0.24	0.31	0.29	0.28	0.38	0.40
***(Z)*-3-hexen-1-ol**	0.32	0.33	0.99	0.28	0.83	1.75	1.00	0.50	0.28	0.20	0.23	0.67	0.24	0.27	0.27	0.54	0.34	0.33
**acetic acid**	nd	nd	nd	nd	nd	nd	nd	nd	nd	nd	nd	nd	nd	nd	nd	nd	0.53	nd
**propionic acid**	nd	nd	nd	nd	nd	0.49	0.35	nd	nd	nd	nd	nd	nd	nd	nd	0.52	0.56	nd
**eucalyptol**	0.92	0.96	1.00	0.57	1.08	1.01	2.94	2.27	0.92	1.96	0.93	1.13	0.85	1.66	1.12	1.29	1.39	2.33
**2-pentyl furan**	0.48	0.59	0.54	0.49	0.79	0.67	1.48	1.24	0.74	0.85	0.62	0.72	0.51	1.06	0.75	0.69	0.70	1.21
**1-methyl pentyl hydroperoxide**	0.50	0.56	0.83	0.49	0.97	1.02	1.18	1.05	0.49	0.67	0.59	0.62	0.56	0.68	0.67	0.70	0.68	0.70

***trans*-linalool oxide**	nd	nd	0.19	nd	nd	0.65	0.38	0.23	0.24	0.30	0.32	0.35	0.32	0.35	0.30	0.57	1.36	0.67

[a] nd: not detected (zero was used as the area for the non-detected compounds during the model's calculation).
